# Variation in gene expression within clones of the earthworm *Dendrobaena octaedra*

**DOI:** 10.1371/journal.pone.0174960

**Published:** 2017-04-06

**Authors:** Marina Mustonen, Jari Haimi, Jenni Kesäniemi, Harri Högmander, K. Emily Knott

**Affiliations:** 1 University of Jyvaskyla, Department of Biological and Environmental Science, University of Jyvaskyla, Jyvaskyla, Finland; 2 University of Oulu, Department of Ecology, University of Oulu, Oulu, Finland; 3 University of Jyvaskyla, Department of Mathematics and Statistics, University of Jyvaskyla, Jyvaskyla, Finland; CSIRO, AUSTRALIA

## Abstract

Gene expression is highly plastic, which can help organisms to both acclimate and adapt to changing environments. Possible variation in gene expression among individuals with the same genotype (among clones) is not widely considered, even though it could impact the results of studies that focus on gene expression phenotypes, for example studies using clonal lines. We examined the extent of within and between clone variation in gene expression in the earthworm *Dendrobaena octaedra*, which reproduces through apomictic parthenogenesis. Five microsatellite markers were developed and used to confirm that offspring are genetic clones of their parent. After that, expression of 12 genes was measured from five individuals each from six clonal lines after exposure to copper contaminated soil. Variation in gene expression was higher over all genotypes than within genotypes, as initially assumed. A subset of the genes was also examined in the offspring of exposed individuals in two of the clonal lines. In this case, variation in gene expression within genotypes was as high as that observed over all genotypes. One gene in particular (chymotrypsin inhibitor) also showed significant differences in the expression levels among genetically identical individuals. Gene expression can vary considerably, and the extent of variation may depend on the genotypes and genes studied. Ensuring a large sample, with many different genotypes, is critical in studies comparing gene expression phenotypes. Researchers should be especially cautious inferring gene expression phenotypes when using only a single clonal or inbred line, since the results might be specific to only certain genotypes.

## 1. Introduction

Gene expression is regulated at multiple stages (transcriptional and post-transcriptional), and is highly plastic, allowing organisms to both acclimate and adapt to changing environmental conditions [1, 2, 3]. For example, genes related to stress tolerance may show wide variation in expression depending on the conditions organisms experience (e.g. [2, 4, 5]). Genes are expressed differently in different tissues, both during ontogenesis [6] and in fully developed adults [7]. Nowadays, measuring gene expression is relatively easy, allowing examination of gene expression levels as a phenotypic response to experimental conditions. Such studies have helped to identify the mechanisms underlying phenotypic changes and to clarify the connection between genotype and phenotype [8, 9]. However, because gene expression can vary, the variation must also be considered when examining gene expression as a phenotypic response. One approach to reduce the possible variation is to examine only a single clonal genotype or nearly identical individuals from inbred lines. An alternative approach is to encompass the possible variation by using pools of different individuals when determining gene expression levels [9].

The amount of variation in gene expression response to identical conditions among individuals sharing the same clonal genotype is rarely reported, but it is often assumed that variation in gene expression among clones will be less than that among individuals with different genotypes. Nevertheless, it is feasible that genotypically identical individuals might not express genes in an identical way [10, 11]. Such variation could be due to physiological differences among the clonal individuals [6, 7] or due to epigenetic differences [12], as shown recently in clonally propagated oil palms [13]. Wolf [14] and Pritchard and colleagues [15] found variation in the gene expression phenotype of mice from a single hybrid inbred line that were exposed to the same conditions. Wolff [14] attributed the differences to possible unrecognized variation in the microenvironment, whereas Pritchard and colleagues [15] concluded that there is tolerance for a wide range of gene expression. Variation in gene expression among individuals from the same clonal genotype might have practical implications for comparative studies that are not widely recognized. Namely, the results of studies focusing on only a single clone or inbred line might not be relevant to other clones or lines, and the results of studies using pools of individuals might mask important variation [9].

An alternative problem also exists: experimental individuals assumed to be clones could actually harbor unexpected genetic differences that affect gene expression. For example, Nota and colleagues [16] found that one of their “clonal” lines of the collembolan *Folsomia candida*, a common test organism for ecotoxicological tests, was actually composed of two genetically different lineages that had surprisingly different expression profiles, with one lineage being much less affected by cadmium exposure than the other. They cautioned that outcomes of tests using *F*. *candida* could lead to different interpretations if possible genetic variation in presumed clonal lines is overlooked [16]. Similarly, Dalziel and colleagues [8] advocated using clonal lineages to control for background genetic variation when studying variation in candidate genes, but cautioned that the major challenge for this approach is the possibility for unrecognized additional mutations within the clonal lineage. In parthenogenetic aphids, high mutation rates have been observed, putting into question the genetic fidelity of clonal lines [10, 11, 17].

This study focuses on gene expression and genetic diversity of the clonally reproducing earthworm, *Dendrobaena octaedra*. This epigeic earthworm is an ecologically important decomposer animal in Northern Boreal forests [18, 19]. *D*. *octaedra* has high genetic diversity [20, 21, 22, 23] and it is able to adapt to metal contaminated habitats [24, 25, 26]. Our previous study revealed that *D*. *octaedra* individuals from two populations differing in metal exposure history had significantly different expression of the metallothionein-2 gene and showed different gene expression responses in an exposure experiment [27]. However, we did not specifically assess genotypic diversity in the samples used in that study. Here, we aim to test if gene expression responses differ among individuals sharing the same clonal genotype when they are exposed to copper contaminated soil.

*D*. *octaedra* is polyploid and expected to produce clonal offspring through apomictic (mitotic) parthenogenesis [28, 29, 30]. Although a deviation from apomictic parthenogenesis is unlikely, Simonsen and Holmstrup [31] found differences among offspring and their parents in allozyme markers, which led them to question whether some mechanism other than apomictic parthenogenesis can be employed by this species. Although allozyme markers can be used to survey genotypic differences among individuals, their detection is based on the activity of the enzymes, and therefore, the variation observed could also reflect phenotypic (gene expression) differences among individuals.

We developed microsatellite markers for an alternative method to genotype individuals of *D*. *octaedra*. The microsatellite markers were used to determine if individuals in cultures of *D*. *octaedra*, initiated with either one or two founding individuals, shared the same genotype (were clones) or if there was evidence for genetic recombination. After determining whether our cultures were reproducing clonally, we used qPCR to examine variation in gene expression both within and among genotypes in two generations of earthworms. We hypothesized that individuals sharing the same genotype would show less variation in gene expression than individuals with different genotypes. It is important to test this assumption and quantify how much variation in gene expression within genotypes exists so that potential causes of the variation, whether they are genetic or epigenetic, can be explored. Furthermore, we estimated and compared gene expression levels to determine if the variation in gene expression would affect our interpretation of the biological significance of the gene expression phenotypes.

## 2. Materials and methods

### 2.1. Sampling

Individuals of *Dendrobaena octaedra* were collected from two locations: a metal contaminated site in Harjavalta (South-West Finland, 61°18'50''N, 22°08'30''E) and an uncontaminated site in Jyväskylä (Central Finland, 62°12'39''N, 25°44'13''E). Earthworms were collected by hand from both sites in September and October 2012 and transferred to the laboratory in buckets with soil from the sampling site. No specific permissions were required for collecting samples since the locations were public areas and the species collected was not endangered. Cultures were then established in the laboratory, coded with a letter (H or J), to indicate the population origin of the founding individuals, and a number. For this study, the population origin of the earthworms was not our primary focus, and individuals from both sites were used to ensure that we obtained different genotypes.

### 2.2. Assessing clonality in *D*. *octaedra*

Juvenile or subadult earthworms collected from the field were used either singly (n = 8) or in pairs (to allow a possibility for mating) (n = 12) to begin cultures of *D*. *octaedra*. Each culture was reared in separate glass jars (Ø 8 cm, with perforated lids) containing uncontaminated organic-rich soil and horse manure (for nutrition). All cultures were maintained at 15°C in a climate cabinet. Soil was changed every few months. *D*. *octaedra* has a long generation time (mean maturation time 21.2 months [32]), and we began monitoring the cultures for cocoons (offspring) after approximately 3 months. The number of cocoons produced by the earthworms in each culture varied. Cocoons were removed from the soil about every 14 days, placed on moist paper towel in lidded Petri dishes and reared at 15°C. The cocoons were monitored for about 90 days and hatched offspring were collected, frozen with liquid nitrogen and stored at -20°C for later DNA extraction and genotyping. Mortality was high, but when possible, the parent earthworm(s) in these cultures were also frozen for later DNA extraction and genotyping after they had produced about 15 cocoons.

#### 2.2.1. Isolation of microsatellite markers

Microsatellite loci were isolated following the FIASCO technique [33] with some modifications (see [34]): the enrichment step included the construction of two microsatellite-enriched libraries using (GA)_20_ and (CAG)_11_ probes. Potential loci were cloned using the pCR2.1-TOPO vector and TOPO-TA cloning kit (Invitrogen) and One Shot TOP10 competent *Escherichia coli* cells. Positive clones were first amplified and sequenced with vector-specific M13 primers. When microsatellite repeats were found in the sequences, new locus specific primers were designed to the flanking regions of the repeat regions using PRIMER3 [35]. Primers were designed for six microsatellite loci, but, after initial testing, one (DO5) did not amplify consistently and was not considered further. The other five loci are described in Table 1.

**Table 1:** Microsatellite loci in *Dendrobaena octaedra*.

Locus name	Repeat	Sequence of F-primer (5’-3’)	Sequence of R-primer (5’-3’)	Expected product	Fluorescent ABI dyes	GenBank Accession (BankIt 1871234)
DO1	(GAAGAGA_3_/GA_11_/GAAA_4_) interrupted	GAGTCAGTTGGACAATTACACTGG	TCTTCCTCTCTTATACATGTAAGTCAA	234	Red (PET)	KU160480
DO2	(GA)_10_	ACATGCTGCTTGGTTCCTTC	AAGCCGATGCACAGGAAAG	160	Blue (6-FAM)	KU160481
DO3	(GACA)_10_	ATGCGGATTATGGAGACCAA	TGCAGCAGTCTTGCTCTTTC	183	Green (VIC)	KU160482
DO4	(GA)_9_	GCCTTAGCGACCGTATTTTG	TCGCATAGCTGTTGTTGCAT	229	Yellow/black (NED)	KU160483
DO6	(GA)_6_(N)_16_(GA)_4_	GTGGTCCTGTTTACTCCCAAG	TCCATCTCTCCATGTTTCCTC	100	Red (PET)	KU160484

#### 2.2.2. Genotyping

Genomic DNA was extracted from either whole earthworms (offspring) or a piece of the anterior end (parents) using Qiagen chemistry (DNeasy kit reagents) and a Kingfisher magnetic processor (Thermo Fisher Scientific). Success of the DNA extractions was confirmed with agarose gel electrophoresis. Amplification was performed separately for each microsatellite locus in 10 μl reactions containing 1 μl of template DNA, 1X buffer (Biotools), 0.4 mM dNTPs (Fermentas), 1 μM reverse primer, 0.9 μM forward primer (TAG Copenhagen), 0.1 μM labeled forward primer (Applied Biosystems), 2 mM MgCl_2_ (Biotools) and 0.5 units Taq polymerase (Biotools). Thermocycling conditions were 94°C for 3 min, then 35 cycles of 94°C for 30 s, 55°C for 30 s, 72°C for 30 s, ending with a final extension of 72°C for 10 min. Amplification products were separated using the ABI PRISM 3130xl with GeneScan 500 LIZ size standard and genotyped using GeneMapper 5 software (all Applied Biosystems). Because *D*. *octaedra* are polyploid (6n), we expected to find variable numbers of alleles (1–6).

### 2.3. Gene expression variation among clones

An additional six cultures, each begun with a single juvenile or subadult individual, were used to provide earthworms for the gene expression study. These cultures were maintained as described earlier, and after about 24 months, five presumably clonal individuals (adult offspring of the wild caught earthworms) were removed from each culture and re-housed separately in Ø 5.5 cm glass jars with perforated lids containing copper contaminated soil for the experiment (see below). An additional earthworm from each culture was used for genotyping (as described above) to ensure that different genotypes were used in the experiment (Supporting information S1 Table).

For the experiment, we wanted to expose the earthworms to conditions that could elicit a gene expression response in the worms, so instead of using the same organic-rich soil used during culturing, we used soil contaminated with copper (100 mg/ kg^-1^ dry mass). Soil used in the experiment was collected from an uncontaminated mature spruce forest in Jyväskylä (soil water content = 52.5%; pH = 5.3; organic matter content = 82.1%). We mixed 191.3 mg of copper chloride (CuCl_2_ H_2_O) in 1500 ml of water thoroughly with 1.5 kg soil (fresh mass) to achieve the intended contamination level. After addition of the copper chloride, pH of the soil decreased to 5.1 and soil water content increased to 76.3%. The spiked soil was incubated for 7 days at 15°C before the experiment. During the experiment, water was added to the cultures every other week to replace what was lost due to evaporation. As we did not intend to study the effect of Cu exposure *per se*, we used only a single Cu concentration in the experiment. Our previous study [27] indicated that this level of Cu contamination was not lethal to *Dendrobaena octaedra*.

**Fig 1:**
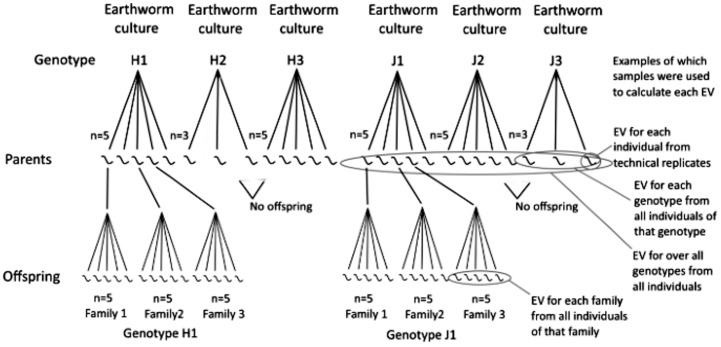
Figure showing the experimental setup. In the parent generation, five individuals per genotype were exposed to copper contaminated soil, except for genotypes H2 and J3, for which there were only three individuals per genotype. In the offspring generation, five offspring each from three individuals in two different genotypes were exposed to copper contaminated soil. The variation in gene expression (EV) was calculated for different groups in the parent and offspring generations, (within individuals, within families, within genotypes and over all genotypes) as shown in the diagram; see section 2.3.2. for more information.

Fig 1 shows the experimental setup. The experiment was begun using five individuals per genotype from six different genotypes (clonal lines reared separately as described above). These worms were the parent generation in the experiment. The parents were exposed to the contaminated soil for two months, during which they produced cocoons. Cocoons were collected from the experimental cultures, placed on moist paper towel in lidded petri dishes, and maintained at 15°C. After the offspring had hatched, they were also reared separately in the Cu contaminated (100 mg/kg dry mass) soil for two months (the offspring generation). After their respective two-month exposure periods, the parent and offspring earthworms were removed from their cultures and put in glass jars with moist paper towel for two days to allow them to empty their guts. Paper towel was changed after one day. A piece of tissue (ca. 5 mm) from the anterior end of each parent earthworm, and about half of each offspring earthworm (also ca. 5 mm) was cut with a scalpel, placed in separate 1.5 ml microcentrifuge tubes and immediately frozen in liquid nitrogen. Samples were stored at -80°C until RNA extraction (approx. 1–2 months).

#### 2.3.1. RNA extraction and RT-qPCR

Our goal was to examine gene expression of each genotype in all of the exposed parents (five per genotype) as well as in five offspring each from three of the exposed parents of each genotype. However, due to some molding of the cocoons, enough offspring hatched only from two of the six genotypes. Therefore, we examined variation in gene expression among exposed parental clones in six genotypes and variation among exposed offspring clones in only two genotypes. There was also some mortality of parents in two of the genotypes during the exposure (H2 and J3, see Fig 1). Twelve target genes were investigated in the parents. The examined genes were chosen to represent a general physiological response of the earthworms to the copper contaminated soil. These included stress-response genes: metallothionein (MT), heat shock protein 40 (HSP40), and heat shock protein 70 (HSP70); as well as genes expected to be involved in metabolism: aldo/keto reductase (AkRed), carbonyl reductase (CarRed), chitinase domain (ChitDo), chymotrypsin inhibitor (ChymInh), dehydrogenase (DeHyd), (similar to) fucosidase (Fuco), leucine aminopeptidase-like protein (Leuc), pyruvate dehydrogenase (Pyr), and L-xylulose reductase (Xyl). We also included analyses of four potential reference genes: 18S-rDNA, 28S-rDNA, peptidylprolyl isomerase (PepIso) and tubulin (Tub). All primers, except those for MT, 18S and 28S (described in [27]), and HSP70 [26], were designed based on a draft transcriptome of *D*. *octaedra* (shared by M. Holmstrup, unpublished, see supporting information S2 Table). A subset of the target genes (AkRed, ChitDo, ChymInh, DeHyd, and MT) were analyzed in the offspring. These were chosen because they showed the most variation in gene expression in the parents.

For RNA extraction, we used the Aurum Total RNA mini kit (Bio-Rad) following the manufacturer’s protocol including *DNase I* treatment. The concentration of the extracted RNA was measured using the Qubit RNA Assay Kit and Qubit Fluorometer (Invitrogen, Turner BioSystems). For cDNA synthesis, we used the iScript cDNA synthesis kit (Bio-Rad) following the manufacturer’s protocol, using 35 ng of RNA per reaction. After synthesis, cDNA was diluted 1:5. Real-time quantitative polymerase chain reactions (qPCR) were performed using IQ SYBR green supermix (Bio-Rad). Prior to analysis, qPCR conditions were optimized and efficiencies were checked with a dilution series (5 points, 10-fold dilutions) (see supporting information S2 Table). In each reaction we used 1 μl of cDNA template and 0.5 μM of each primer in a final reaction volume of 20 μl (to estimate efficiency) or 10 μl (for the gene expression comparisons). Three replicate reactions for each sample using the same cDNA preparation were prepared and an inter-run calibrator was used. For all reactions, we used a CFX96 C1000 Touch Thermal Cycler (Bio-Rad) with the following protocol: 94°C for 2 minutes and then 40 cycles of 94°C for 30 sec, 60°C for 30 sec and 72°C for 15 sec followed by a plate read. A melt curve analysis was done at the end of each amplification reaction, and melt curves were checked to ensure that there was only a single PCR-product.

#### 2.3.2. Data analysis

Inter-run calibration was done with GenEx (version 6.1, MultiD Analyses) and the calibrated data and stability of the reference genes was checked with qBase+ (Biogazelle). Data from failed reactions (Cq > 35) and outlying technical replicates (having more than 1 cycle difference in Cq in comparison to the other technical replicates) were removed since these represent technical artifacts introduced during preparation of the qPCR reactions (e.g. pipetting errors). Based on our previous study [27], we expected that 18S and 28S rRNA genes would be useful reference genes. However, even though these genes showed stable expression in the parent dataset, their expression was too variable in the offspring dataset (data not shown). Two other potential reference genes were tested (PepIso and Tub), but expression of these genes also varied among the samples (in both parent and offspring datasets). Therefore, we normalized the Cq -values with NORMA-Gene [36], which utilizes the entire dataset (target and potential reference genes) to reduce systematic and artificial between-replicate bias with a least squares method. NORMA-gene has been shown to be reliable even with small datasets and to outperform normalization by reference genes, but requires that a block design is used to reduce variation between treatments [36]. Thus, it provides a reliable normalization method for our study.

In order to test the assumption that there is less variation in gene expression among clonal individuals than among individuals with different genotypes, we used the normalized Cq-values to calculate estimates of variation within different groups (within individuals, within genotypes, and over all genotypes, and for the offspring dataset also within families). The estimate of variation (EV) was calculated from the sample’s variance which was adjusted with the corresponding degrees of freedom to allow comparison of the differently sized groups:

$$EV=\frac{\sum \text{variance * }\left(\text{Nx}-1\right)}{\text{Na}-\text{G}}$$

∑variance = sum of the sample variance for the group for which EV is calculated (EV is calculated for each target gene separately)

Nx = number of observations in the group for which EV is calculated

Na = number of all observations

G = number of the groups for which EV is calculated

EV within individuals was calculated from the normalized Cq-values of technical replicates. This value allows us to visualize how much variation is due to uncontrollable technical variation and is not biologically relevant. EVs within genotypes were calculated from normalized Cq-values of all individuals having the same genotype (for parent and offspring datasets separately). Similarly, we calculated EV within “families” from the normalized Cq-values of all offspring from a single parent (for the offspring dataset only). Finally, EV over all genotypes was calculated from normalized Cq-values from all samples, both for the parent dataset and the offspring dataset. All EV- values were calculated for each gene separately.

We then evaluated whether there were differences in the estimates of variation (EV) between the different groups (within individuals, within families, within genotypes, and over all genotypes). There were 12 genes in the parent generation and five genes in the offspring generation; and additionally we compared the same subset of five genes in both datasets (parents and offspring). First, using data from all target genes, the comparisons were made using repeated measures ANOVA with gene as the repeated factor (repeated measures ANOVA was chosen in order to take the dependence in the dataset into account.). Pair-wise comparisons were done with LSD post hoc test. Second, comparisons were done for each gene separately using Bartlett’s test to see if the variation in EV between the different groups showed the same pattern for all genes, and pair-wise comparisons were done using the F-test. We also tested whether there were significant differences in EV between different genotypes (in parents) and between different families (in the offspring dataset) with repeated measures ANOVA. Differences between EV of parents and offspring with the same genotype were tested with the Mann-Whitney U test. The signed rank test was chosen as a robust (against non-normality) method for comparison of two related (here, gene-wise) samples.

In addition to variation in gene expression (EV), the levels of gene expression were compared, using relative quantity (RQ) values calculated in qBase+. Relative Quantity values are linear transformations of the Cq-values converted with the efficiencies of the qPCR reactions taken into account [37] that express gene expression as relative to another value (in this case, average Cq over all samples). As for the EV-values, these were corrected for inter-run calibration and normalized using NORMA-gene [36]. Normalized RQ-values were used to compare gene expression levels between offspring of the same genotype (but from different parents) i.e. between “families”, between parents and offspring of the same genotype, and between different genotypes (H1, H2, H3, J1, J2 and J3). The Mann-Whitney test was used when comparing two groups (offspring to parents) and the Kruskal-Wallis test was used in cases of more than two groups (different genotypes or families). All statistical analyses were carried out in Excel and in IBM Statistics SPSS 20.

## 3. Results

### 3.1. Clonal reproduction

Altogether 170 individuals were genotyped. Genotypes were checked from eight single-worm cultures and 12 pair-worm cultures. Not all parent individuals survived in culture until DNA extraction and there were varying numbers of offspring per culture. Supporting information S3 Table details all individuals and their multi-locus genotypes (allele sizes). Of the five microsatellite loci amplified, one (DO4) was monomorphic. Altogether 15 different multi-locus genotypes were observed. We found only one multi-locus genotype in each of the single-worm cultures: all earthworms from these cultures had the same allele sizes, including the parent earthworm when it was available for comparison. One or two multi-locus genotypes were found in each of the pair-worm cultures. Multi-locus genotypes from the offspring in these cultures matched one or the other of the founding parents (when comparison was possible). One of the multi-locus genotypes (e.g. H6, see Supporting information S3 Table) was most common, and was found in half of the single-worm cultures and approximately one-third of the pair-worm cultures.

### 3.2. Gene expression variation

**Fig 2:**
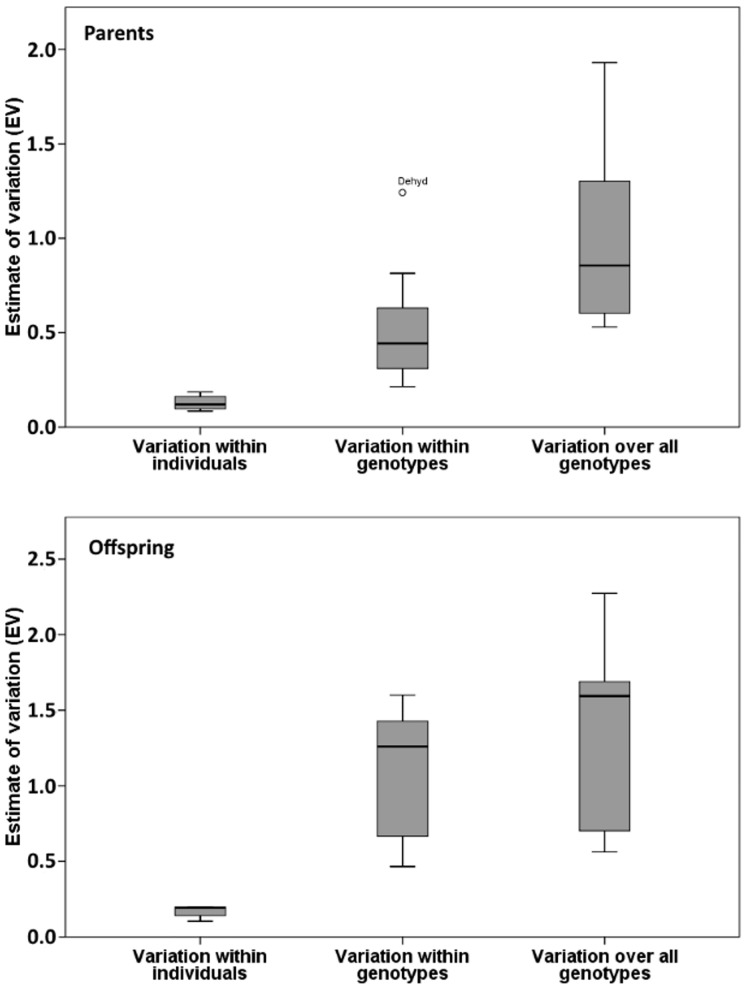
Estimate of Variation (EV) in the different groups (within individuals, within genotypes and over all genotypes) in both the parent and offspring datasets. Box plot shows all the EV values calculated for each gene separately for each of the groups (median, 25% upper and lower quartile, minimum, maximum, and outliers).

We examined variation in the expression of 12 or five target genes (in parents and offspring, respectively) and compared the extent of variation within genotypes and over all genotypes. Combining data from all genes, the estimates of variation (EV) differed significantly between the different groups in the study design (within individuals, within genotypes, and over all genotypes) both in the parent dataset (repeated measures ANOVA, F = 33.853, df_1_ = 2, df_2_ = 22, p < 0.001), and in the offspring dataset (F = 13.229, df_1_ = 2, df_2_ = 8, p = 0.003). Variation increased from within individuals to within genotypes to over all genotypes in both datasets (Fig 2). Pair-wise comparisons revealed that in the parent dataset there were significant differences in variation between all groups, whereas in the offspring dataset a significant difference was found only in comparisons to the technical variation (EV within individuals); EV within genotypes and EV over all genotypes did not differ from each other (Table 2). When taking into consideration the additional level of “within families” in the offspring dataset, the estimates of variation (EV) differed significantly between the different groups in the study design (within individuals, within families, within genotypes, and over all genotypes, F = 10.284, df_1_ = 3, df_2_ = 12, p = 0.001), and the pair-wise comparisons showed that there was a significant difference between within individuals and the other groups, but not between within families, within genotypes and over all genotypes (Supporting information S4 Table, Supporting information S1 Fig). When analyzing the same subset of genes in the parent data as in the offspring data (five genes), a significant difference was found between the groups (F = 10.614, df_1_ = 2, df_2_ = 8, p = 0.006), but the difference was significant only between within individuals and within genotypes (LSD, mean difference = 0.526, p = 0.032) and not between EV within genotypes and EV over all genotypes (LSD, mean difference = 0.462, p = 0.051), although the difference is nearly significant (Supporting information S2 Fig). When analyzing the genes separately, a significant difference in EV between the groups was found for all, but pair-wise comparisons revealed no significant differences within genotypes and over all genotypes in some genes (AkRed, ChitDo, Dehyd and HSP40 and HSP70) in the parent dataset, and all genes except ChymInh in the offspring dataset; Supporting information S5 Table).

**Table 2:** Differences in the Estimate of Variation values (EV) between each group in the study design in both in the parent (12 genes) and offspring datasets (five genes). Pair-wise comparisons for differences in EV were performed using LSD post hoc test.

		Within individuals		Within genotypes	
		Mean difference	p value	Mean difference	p value
Parents	Within genotypes	0.388	0.001	-	-
	Over all genotypes	0.861	<0.001	0.473	0.001
Offspring	Within genotypes	0.917	0.014	-	-
	Over all genotypes	1.197	0.020	0.280	0.157

In the parent dataset, the six different genotypes included in the study did not differ significantly in EV (F = 1.021, df_1_ = 2.827, df_2_ = 31.099, p = 0.393; Greenhouse-Geisser correction of df_2_ was used due to non-sphericity, ε = 0.565; Supporting information S3 Fig). The same was observed when analyzing only the subset of five genes from the parent data (data not shown). In the offspring dataset, there was higher EV in genotype J1 than in genotype H1 (Z = 2.310, p = 0.021) (Supporting information S4 Fig). There was no significant difference between the EVs of different families (F = 2.283, df_1_ = 2.489 df_2_ = 9.956, p = 0.147; Greenhouse-Geisser correction of df_2_ was used due to non-sphericity, ε = 0.498).

**Fig 3:**
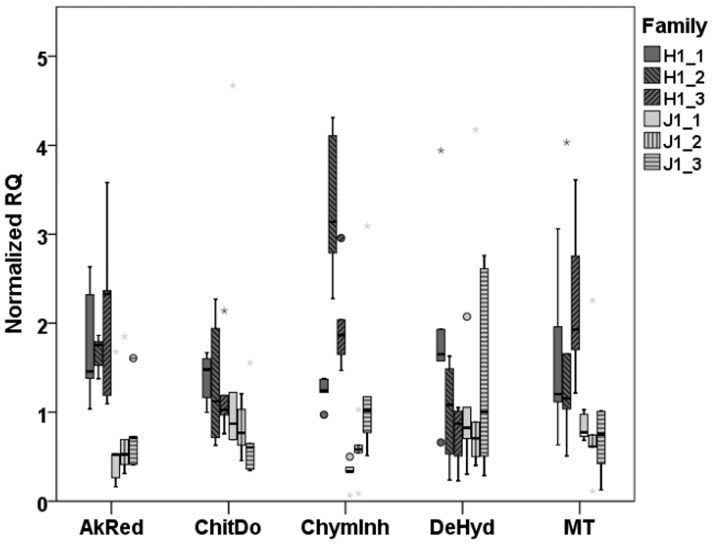
Gene expression (RQ) in offspring of each family. The box plot shows all the individuals of the family (median, 25% upper and lower quartile, minimum, maximum, and outliers).

Despite the fact that variation in gene expression (EV) was similar among the different genotypes in the parent dataset, when comparing gene expression levels (RQ), the different genotypes showed differences in the expression of most genes (Supporting information S6 Table). In these cases the differences were mostly due to one genotype (J3) having higher expression than the other genotypes (Supporting information S5 Fig). RQ was also significantly different for some genes between the genotypes in the offspring dataset, but these were not necessarily the same as those showing differences among parental genotypes (Supporting information S6 Table, Fig 3).

A difference in gene expression (RQ) levels among families was observed in one gene out of five. Offspring from different parents (but sharing the same genotype) showed a significant difference in the expression of ChymInh, which was noted in both offspring genotypes, H1 (Kruskal-Wallis, χ^2^ = 11.58, df = 2, p = 0.003) and J1 (χ^2^ = 8.06, df = 2, p = 0.018; Fig 3). Gene expression of offspring differed from that of parents with the same genotype in H1 (all offspring of genotype H1 compared to all parents of genotype H1) in two genes: ChymInh (Mann-Whitney, U = 6.00, df = 1, p = 0.004) and MT (U = 3.00, df = 1, p = 0.001) and in genotype J1 in one gene: ChymInh (Mann-Whitney, U = 10.00, df = 1, p = 0.015).

## 4. Discussion

Given that gene expression is noisy [3] and potentially under the influence of epigenetic regulation, assuming that clonally propagated individuals are genetically identical and that they will show a similar gene expression response to experimental conditions is probably unrealistic. We developed microsatellite markers to determine whether the earthworm *Dendrobaena octaedra* produces clonal offspring, as expected for an organism with apomictic parthenogenesis, and then we measured gene expression variation both within and among clonal genotypes. Our results show that variation in gene expression within genotypes is greater than that within individuals (greater than technical variation), but can be either lower or similar to the variation observed when analyzing different genotypes. Variation in gene expression within clones can also reflect different gene expression responses of “identical individuals” exposed to the same conditions, but this might be dependent upon the specific genotype and the genes being investigated. Our results suggest that comparative studies of gene expression phenotypes could be affected by variation within genotypes. Some genotypes show more variation than others, so results could be biased by what genotypes happen to be included in the sample or they could be specific to a single genotype only.

One explanation for finding variation in gene expression within a single clonal genotype could be that there is unidentified genetic variation within the sample and that the individuals are not really clones. In our experiment, there is a possibility that mutations have occurred during the culturing time (1–2 generations) and that the individuals are genetically different despite originating from a single parent. Nota and colleagues [16] showed that there can be very distinct differences in gene expression between different genotypes of *Folsomia candida* originating from the same clonal line. Similarly, Dudycha and colleagues [9] found differences in gene expression between different clones within *Daphnia* ecotypes. In clonal aphid lineages, mutations can cause rapid changes in the lineages, giving rise to phenotypic variation that is subject to selection [10, 11, 17]. Genotyping with the microsatellite markers described here confirmed that individuals in our cultures shared the same multi-locus genotype: identical allele sizes were found among individuals reared in cultures begun with a single founder worm. In cultures begun with a pair of founding individuals, either 1 or 2 multi-locus genotypes were found. However, this conclusion is necessarily limited by the number of genetic markers used in genotyping. It might be that genetic differences among individuals were not surveyed or were overlooked, and our conclusions would be stronger if more genetic markers had been used. Nevertheless, there is strong evidence for apomictic parthenogenesis in this species [28, 29, 30]. And, even though Simonsen and Holmstrup [31] found some differences between offspring and their parent in allozyme markers, most parents and offspring in their study were identical, and the variation they observed could also have derived from variation in gene expression.

In the parent generation we found lower EV within genotypes than over all genotypes. Even though there was significant variation in gene expression among individuals sharing the same genotype (compared to technical variation), genetically identical individuals were more similar in their gene expression than were individuals that were genetically different as is typically assumed. In the offspring dataset, on the other hand, and when we analyzed only a subset of the genes in the parent dataset, the variation in gene expression within genotypes was as high as the variation over all genotypes. Variation was high even within families (among offspring that were from the same parent). This result could be due to our choice of the five genes to be analyzed in the offspring dataset, which were chosen specifically because they showed more variation than other genes in the parental dataset. We might have found a different result if data for all 12 genes had been collected for the offspring.

We observed differences in gene expression (RQ) between offspring and their parents in this study, and among offspring in the same family. Previous work has shown that gene expression can change drastically during adolescence, e.g. in *Caenorhabditis elegans* [38], and variation in gene expression is also known to increase in aging animals [39, 40]. Difference in age and physiology of the two month old juveniles compared to the mature adults in our experiment could explain the difference in gene expression. Carry-over effects from previous experience of the parents might be one explanation: parents were first raised to adulthood in uncontaminated organic rich soil and then exposed to copper contamination, whereas offspring were only exposed to contaminated soil (after hatching). Another possible reason for differences between the offspring and their parents could be that the tissues used in RNA extraction were different: in the parents only a small part from the anterior end was used and in the offspring about half of the earthworm was used for RNA extraction. In the offspring samples there was likely a larger variety of tissue types, which could have led to more variation, since different tissues express genes differently [6, 7]. The difference in tissue composition between parents and offspring could also explain why our potential reference genes (18S and 28S rRNA) were suitable for the parent dataset, but not the offspring dataset.

Variation in gene expression could be beneficial to organisms when they are exposed to changing conditions, and in evolutionary adaptation in the long run [1, 2, 3, 11, 17, 41]. Genes related to stress have been found to show more variation than other genes that have basic cell maintenance functions [42]. In our study, the three stress related genes, HSP40, HSP70, and MT showed high variation within genotypes, but not necessarily more variation than did many of the genes related to metabolism. One gene, ChymInh, which showed significant gene expression differences within genotypes, is an inhibitor of the digestive enzyme chymotrypsin, but it has also been shown to be involved in an immune response [43]. Thus, it is possible that it is a stress related gene of sorts in this species as well.

Determining the mechanisms behind the gene expression variation among individuals sharing the same genotype is beyond the scope of this study. Some possible explanations can be found from studies on cell-to-cell -variation in gene expression [41]. For example, transcriptional bursts in gene expression, when genes transition between transcriptional activity and inactivity due to chromatin remodeling [41], might be one mechanism. Environmental differences could still play a part: despite mixing the Cu in the experimental soil thoroughly, there could have been slightly different concentrations of Cu in the soil and other small differences in the conditions that might have led to different gene expression responses since *D*. *octaedra* are known to avoid patches of soil with highest contamination levels [44]. Micro-environmental variation has been proposed for explanations of gene expression variation among mice from inbred lines [14] and we cannot exclude possible micro-habitat differences, for example in microbial communities. Quality of ingested food could also be varying, which could lead to differences in expression of the genes related to digestion. Epigenetic regulation could be another possible explanation for our results. DNA methylation occurs in earthworms in association with metal tolerance [45]. Whether DNA methylation occurs in *D*. *octaedra* and, if so, whether DNA methylation affects gene expression in this species is not known.

## 5. Conclusions

Studies comparing gene expression phenotypes should take into account the considerable variation in gene expression that can exist even between clonally produced individuals. Variation within genotypes may be significant, as large as that observed among genotypes. Thus, many different genotypes should be used to get a reliable average of the effect (e.g. metal exposure) on gene expression. This is especially important for e.g. ecotoxicological studies, when conclusions are often based on the results from study of a single clonal or inbred line. When using clonal lines, it should be confirmed that individuals truly are genetically identical [10], and even when they are, gene expression should not be assumed to be identical. More research into what causes differences in gene expression among clones and the practical implications of this variation is needed.

## Supporting information

S1 Table Allele sizes of microsatellite loci DO1, DO2, DO3, DO4 and DO6 from *Dendrobaena octaedra* earthworms from each of the cultures used in the gene expression experiment. (PDF)

S2 Table Primer sequences used in qPCR and reaction efficiencies. Transcripts used to design the primers are available on request from Dr. Martin Holmstrup. MT, 18S and 28S are described in Mustonen et al. 2014, and HSP70 in Fisker et al. 2013.
(PDF)

S3 Table Allele sizes of microsatellite loci DO1, DO2, DO3, DO4 and DO6 for *Dendrobaena octaedra* earthworm families originating either from Harjavalta (H) or Jyväskylä (J). Each earthworm culture had originally either one (P1) or two parent earthworms (P1 and P2) which produced offspring (F1). DO4 was monomorphic and not used in genotyping some samples, and DO1 had low signal and could not be scored in some samples (marked with—in the table). The parent individual was not always available for genotyping because of high mortality (marked “Not available” in the table). Shading delineates different clonal lines.
(PDF)

S4 Table Differences in the Estimate of Variation values (EV) between each group in the study design, including the within families group, in the offspring dataset. Pairwise comparisons for differences in EV were performed using LSD post hoc test.
(PDF)

S5 Table Difference in the Estimate of Variation (EV) between different groups (within individuals, within families [in offspring data only], within genotypes and over all genotypes) for each gene separately, and pairwise comparisons between groups: 1. comparison is between within individuals and within families, 2. comparison is between within families and within genotypes for offspring data and within individuals and within genotypes for parent data, and 3. comparison is between within genotypes and over all genotypes. Bartlett test was used for the overall analysis and a F-test was used for pairwise comparisons.
(PDF)

S6 Table Differences in gene expression of each gene between different genotypes. Kruskal Wallis analysis for the parents and Mann-Whitney test for the offspring.
(PDF)

S1 Fig Estimate of Variation (EV) in the different groups (within individuals, within families, within genotypes and over all genotypes) in the offspring dataset. Box plots show the median EV for all target genes (n = 5) and 25% upper and lower quartiles as well as minimum and maximum.
(PDF)

S2 Fig Estimate of Variation (EV) in the different groups (within individuals, within genotypes and over all genotypes) for the parent data when using only the subset of five genes (AkRed, ChitDo, ChymInh, Dehyd and MT). Box plot shows all the EV values calculated for each gene separately for each of the groups (median, 25% upper and lower quartile, minimum, maximum, and outliers).
(PDF)

S3 Fig Estimate of variation in all genes (AkRed, CarRed, ChitDo, ChymInh, DeHyd, Fuco, Leuc, Pyr, Xyl, HSP40, HSP70 and MT) within parent genotypes (H1, H2, H3, J1, J2 and J3). Box plot shows all the EV values calculated for each gene separately for each of the genotypes (median, 25% upper and lower quartile, minimum, maximum, and outliers).
(PDF)

S4 Fig Estimate of Variation (EV) for two genotypes, H1 and J1, in the offspring dataset. Box plot shows all the EV values calculated for each gene separately for each genotype (median, 25% upper and lower quartile, minimum, and maximum).
(PDF)

S5 Fig Gene expression (RQ) of each gene in each parent genotype. Box plot shows all the normalized relative quantity (RQ) values for each genotype (median, 25% upper and lower quartile, minimum, maximum, and outliers).
(PDF)
