# Supplementary material for: Variation in gene expression within clones of the earthworm Dendrobaena octaedra
Source: PLoS One. 2017 Apr 6;12(4):e0174960. doi: 10.1371/journal.pone.0174960 (PMC5383104; doi:10.1371/journal.pone.0174960)
Supplement: S1 Table — (PDF) [file pone.0174960.s001.pdf]

S1 Table. Allele sizes of loci DO1, DO2, DO3, DO4 and DO6 from *Dendrobaena octaedra* earth-worms from each of the cultures used in the gene expression experiment.

Culture	DO1	DO2	DO3	DO4	DO6
H1	231/237	140/153	165/173/177	211/213	96/100
H2	231	140	173/181/189	211/213	96
H3	229/231/233/241/245/253	140/147/151	177/185/187/193	211/213	90/96
J1	228/234/238/240/244	140/153	173/177/193	211/213	90/96
J2	228/234/236/238/240	140/147/151	177/185/187/192	211/213	90/96
J3	228/240	140/157	177	211/213	96/98
