# Supplementary material for: Variation in gene expression within clones of the earthworm Dendrobaena octaedra
Source: PLoS One. 2017 Apr 6;12(4):e0174960. doi: 10.1371/journal.pone.0174960 (PMC5383104; doi:10.1371/journal.pone.0174960)
Supplement: S2 Table — Transcripts used to design the primers are available on request from Dr. Martin Holmstrup. MT, 18S and 28S are described in Mustonen et al. 2014, and HSP70 in Fisker et al. 2013. (PDF) [file pone.0174960.s002.pdf]

S2 Table. Primer sequences used in qPCR and reaction efficiencies. Transcripts used to design the primers are available on request from Dr. Martin Holmstrup[1]. MT, 18S and 28S are described in Mustonen et al. 2014[2], and HSP70 in Fisker et al. 2013[3]

Gene	Primer (5'-3')	Efficiency %
MT-2	F: ACACTCAGTGCTGTGGCAGCG<br>R: GGCTGCGCACTTGCAGGC	103,5
HSP40	F: ATGTCGGCTGGGATGTTGTT<br>R: AAGAAAGGATGGAAGCCGGG	99,8
HSP70	F: AGCTGAGCATCGAGGAGAAG<br>R: TCCTTGAAGTCCTCCACGTC	105,6
AkRed	F: GCTGATCGAGGCAGATCCTT<br>R: CCAAGTCCACGAACCCTGAA	96,2
CarRed	F: ATGGCAAGGATTCAGCAGGG<br>R: TGGGGTAGAAACTCCCTCCG	97,1
ChitDo	F: GAGAGCCAGTCGTGTTTGGA<br>R: GAAACTGCAATGGGACGCAG	94,9
ChymInh	F: TCAGCTACCCGGTTCTAGGT<br>R: AAGAGGCCAAAGCACAGGTT	94,8
DeHyd	F: CTGGAAGAGGAAGACCTGGAT<br>R: GACGCTTACGAACACGCTCT	100,9
Fuco	F: ACGCAAGACCACTTCCTACG<br>R: CCCAGGACGGAAATTCTGCT	95,35
Leuc	F: AGCCACAGGAGTCTTCACCAA<br>R: TGCGCCACCATATCTTCCTG	99,4
Pyr	F: CTGGAGCAGTCAGGCATCAA<br>R: GCCTTGCTTTCCATGATCCG	97,1
Xyl	F: ACATGGCTTTGGAGCTTGGA<br>R: ACGGTCATGCCACCATCAAT	109,8
PepIso	F: CGAAGATGCTGGTTCCTCCT<br>R: CACCGCGTCGTTAGGGATTT	90,6
Tub	F: GCCTCGACAACTTCCGATTC<br>R: ATGGAATTCACCGAGGCTGA	98,9
18S	F: ACCACATCCAAGGAAGGCAG<br>R: CCCGAGATCCAACTACGAGC	93,3
28S	F: TGGTGGAGGTCCGCAGCGAT<br>R: CGTTTCGTCCCCAAGGCCTC	90,5

References

1. Dr.Martin Holmstrup, Aarhus University, Vejsovej 25, building B3.16, 8600 Silkeborg, Denmark. Email: martin.holmstrup@bios.au.dk, phone: +4587158823.

2. Mustonen, M., Haimi, J., Väisänen, A., Knott, K.E. Metallothionein gene expression differs in earthworm populations with different exposure history. Ecotoxicology 2014; 23:1732-1743.

3. Fisker, K.V., Holmstrup, M., Sørensen, J.G. (2013) Variation in metallothionein gene expression is associated with adaptation to copper in the earthworm *Dendrobaena octaedra*. Comp Biochem Physiol C. 2013; 157:220-226.
