# Supplementary material for: Variation in gene expression within clones of the earthworm Dendrobaena octaedra
Source: PLoS One. 2017 Apr 6;12(4):e0174960. doi: 10.1371/journal.pone.0174960 (PMC5383104; doi:10.1371/journal.pone.0174960)
Supplement: S3 Table — Each earthworm culture had originally either one (P1) or two parent earthworms (P1 and P2) which produced offspring (F1). DO4 was monomorphic and not used in genotyping some samples, and DO1 had low signal and could not be scored in some samples (marked with—in the table). The parent individual was not always available for genotyping because of high mortality (marked “Not available” in the table). Shading delineates different clonal lines. (PDF) [file pone.0174960.s003.pdf]

Table 3. Allele sizes of microsatellite loci DO1, DO2, DO3, DO4 and DO6 for *Dendrobaena octaedra* earthworm families originating either from Harjavalta (H) or Jyväskylä (J). Each earthworm culture had originally either one (P1) or two parent-earthworms (P1 and P2) which produced offspring (F1). DO4 was monomorphic and not used in genotyping some samples, and DO1 had low signal and could not be scored in some samples (marked with - in the table). The parent individual was not always available for genotyping because of high mortality (marked "Not available" in the table). Shading delineates different clonal lines.

Single earthworm cultures					
Individual	DO1	DO2	DO3	DO4	DO6
H6P1	231	140	173/181/187	211/213	96
H6F1.1	231	140	173/181/187	211/213	96
H6F1.2	231	140	173/181/187	211/213	96
H6F1.3	231	140	173/181/187	211/213	96
H6F1.4	231	140	173/181/187	211/213	96
H7P1	Not available				
H7F1.2	231	140	173/181/187	211/213	96
H7F1.3	231	140	173/181/187	211/213	96
H7F1.4	231	140	173/181/187	211/213	96
H7F1.5	231	140	173/181/187	211/213	96
H7F1.6	231	140	173/181/187	211/213	96
H7F1.7	231	140	173/181/187	211/213	96
H8P1	217/225/233/241	138/140	165/177/185/193	211/213	96/110
H8F1.1	217/225/233/241	138/140	165/177/185/193	211/213	96/110
H8F1.2	217/225/233/241	138/140	165/177/185/193	211/213	96/110
H8F1.3	217/225/233/241	138/140	165/177/185/193	211/213	96/110
H8F1.4	217/225/233/241	138/140	165/177/185/193	211/213	96/110
H8F1.5	217/225/233/241	138/140	165/177/185/193	211/213	96/110
H8F1.6	217/225/233/241	138/140	165/177/185/193	211/213	96/110
H8F1.7	217/225/233/241	138/140	165/177/185/193	211/213	96/110
H8F1.8	217/225/233/241	138/140	165/177/185/193	211/213	96/110
H8F1.9	217/225/233/241	138/140	165/177/185/193	211/213	96/110
H8F1.10	217/225/233/241	138/140	165/177/185/193	211/213	96/110
H8F1.11	217/225/233/241	138/140	165/177/185/193	211/213	96/110
H8F1.12	217/225/233/241	138/140	165/177/185/193	211/213	96/110
H9P1	219/229/239/248	140/151/173	165/173/177/187/190/193	211/213	96
H9F1.1	219/229/239/248	140/151/173	165/173/177/187/190/193	211/213	96
H9F1.2	219/229/239/248	140/151/173	165/173/177/187/190/193	211/213	96
H9F1.3	219/229/239/248	140/151/173	165/173/177/187/190/193	211/213	96
H12P1	231	140	173/181/187	211/213	96
H12F1.1	231	140	173/181/187	211/213	96
H12F1.2	231	140	173/181/187	211/213	96
H12F1.3	231	140	173/181/187	211/213	96
H12F1.4	231	140	173/181/187	211/213	96
H12F1.5	231	140	173/181/187	211/213	96
H12F1.6	231	140	173/181/187	211/213	96
H13P1	Not available				
H13F1.1	231/235	140/147	165/173/177	211/213	96/100
H13F1.2	231/235	140/147	165/173/177	211/213	96/100

H13F1.3	231/235	140/147	165/173/177	211/213	96/100
H13F1.4	231/235	140/147	165/173/177	211/213	96/100
H14P1	231	140	173/181/187	211/213	96
H14F1.1	231	140	173/181/187	211/213	96
H14F1.2	231	140	173/181/187	211/213	96
H14F1.3	231	140	173/181/187	211/213	96
H14F1.4	231	140	173/181/187	211/213	96
H14F1.5	231	140	173/181/187	211/213	96
H14F1.6	231	140	173/181/187	211/213	96
H14F1.7	231	140	173/181/187	211/213	96
H15P1	221/227/231/235	138/140/171	165/173/177/185	211/213	96/100
H15F1.1	221/227/231/235	138/140/171	165/173/177/185	211/213	96/100
H15F1.2	221/227/231/235	138/140/171	165/173/177/185	211/213	96/100
H15F1.3	221/227/231/235	138/140/171	165/173/177/185	211/213	96/100
H15F1.4	221/227/231/235	138/140/171	165/173/177/185	211/213	96/100
H15F1.5	221/227/231/235	138/140/171	165/173/177/185	211/213	96/100
H15F1.6	221/227/231/235	138/140/171	165/173/177/185	211/213	96/100
H15F1.7	221/227/231/235	138/140/171	165/173/177/185	211/213	96/100
H15F1.8	221/227/231/235	138/140/171	165/173/177/185	211/213	96/100
H15F1.9	221/227/231/235	138/140/171	165/173/177/185	211/213	96/100
H15F1.10	221/227/231/235	138/140/171	165/173/177/185	211/213	96/100
H15F1.11	221/227/231/235	-	165/173/177/185	211/213	96/100
H15F1.12	221/227/231/235	138/140/171	165/173/177/185	211/213	96/100
H15F1.13	221/227/231/235	138/140/171	165/173/177/185	211/213	96/100
H15F1.14	221/227/231/235	138/140/171	165/173/177/185	211/213	96/100
H15F1.15	221/227/231/235	138/140/171	165/173/177/185	211/213	96/100

Pair earthworm cultures

Individual	DO1	DO2	DO3	DO4	DO6
H112P1	217/225/231/233	138/140	165/177/185/193	211/213	96/100
H112P2	Not available				
H112F1.1	231/233/235	140/147	165/173/177	211/213	96/100
H114P1	Not available				
H114P2	Not available				
H114F1.1	231	140	173/181/187	211/213	96
H114F1.2	231	140	173/181/187	211/213	96
H114F1.3	231	140	173/181/187	211/213	96
H114F1.4	231	140	173/181/187	211/213	96
H114F1.5	231	140	173/181/187	211/213	96
H116P1	231	140	173/181/187	211/213	96
H116P2	231	140	173/181/187	211/213	96
H116F1.1	231	140	173/181/187	211/213	96
H116F1.2	231	140	173/181/187	211/213	96
H118P1	231	140	173/181/187	211/213	96
H118P2	Not available				
H118F1.1	231	140	173/181/187	211/213	96
H118F1.2	-	140	173/181/187	211/213	96
H118F1.3	-	140	173/181/187	211/213	-
H118F1.4	231	140	173/181/187	211/213	96
H118F1.5	231	140	173/181/187	211/213	96
H118F1.6	-	140	173/181/187	211/213	96
H120P1	-	138/140	165/173/177/185	211/213	96/100

H120P2	Not available				
H120F1.1	-	140/153/155/159	173/177/186/190	211/213	96/100
H120F1.2	-	140/153/155/159	173/177/186/190	211/213	96/100
H120F1.3	-	140/153/155/159	173/177/186/190	211/213	96/100
H120F1.4	-	140/153/155/159	173/177/186/190	211/213	96/100
H120F1.5	-	140/153/155/159	173/177/186/190	211/213	96/100
H120F1.6	-	140/153/155/159	173/177/186/190	211/213	96/100
H120F1.7	-	140/153/155/159	173/177/186/190	211/213	96/100
H120F1.8	-	140/153/155/159	173/177/186/190	211/213	96/100
H120F1.9	-	140/153/155/159	173/177/186/190	211/213	96/100
H120F1.10	-	140/153/155/159	173/177/186/190	211/213	96/100
H120F1.11	-	140/153/155/159	173/177/186/190	211/213	96/100
H120F1.12	221/227/231/235	138/140	165/173/177/185	211/213	96/100
H120F1.13	-	140/153/155/159	173/177/186/190	211/213	96/100
H120F1.14	-	140/153/155/159	173/177/186/190	211/213	96/100
H120F1.15	-	140/153/155/159	173/177/186/190	211/213	96/100
H120F1.16	221/227/231/235	138/140	165/173/177/185	211/213	96/100
H120F1.17	-	138/140	165/173/177/185	211/213	96/100
H120F1.18	221/227/231/235	138/140	165/173/177/185	211/213	96/100
H120F1.19	221/227/231/235	138/140	165/173/177/185	211/213	96/100
H120F1.20	221/227/231/235	138/140	165/173/177/185	211/213	96/100
H120F1.21	-	140/153/155/159	173/177/186/190	211/213	96/100
H120F1.22	221/227/231/235	138/140	165/173/177/185	211/213	96/100
H120F1.23	221/227/231/235	138/140	165/173/177/185	211/213	96/100
H121P1	231/233/235	140/147	165/173/177	211/213	96/100
H121P2	Not available				
H121F1.1	231	140	173/181/187	211/213	96
H121F1.2	231	140	173/181/187	211/213	96
H121F1.3	231	140	173/181/187	211/213	96
H201P1	-	140	173/181/187	-	96
H201P2	Not available				
H201F1.1	230/232/234/236/238/246	140/147	165/173/177	-	96
H201F1.2	-	140/147	165/173/177	-	96
H201F1.3	228/230/232/234/236/238	140	173/181/187	-	96
H201F1.4	228/230/232/234/236/238	140	173/181/187	-	96
H201F1.5	228/230/232/234/236/238	140	173/181/187	-	96
H201F1.6	228/230/232/234/236/238	140	173/181/187	-	96
H201F1.7	228/230/232/234/236/238	140	173/181/187	-	96
H201F1.8	228/230/232/234/236/238	140	173/181/187	-	96
H201F1.9	228/230/232/234/236/238	140	173/181/187	-	96
H201F1.10	228/230/232/234/236/238	140	173/181/187	-	96
H201F1.11	228/230/232/234/236/238	140	173/181/187	-	96
H201F1.12	228/230/232/234/236/238	140	173/181/187	-	96
H201F1.13	228/230/232/234/236/238	140	173/181/187	-	96
H201F1.14	228/230/232/234/236/238	140	173/181/187	-	96
H201F1.15	228/230/232/234/236/238	140	173/181/187	-	96
H201F1.16	228/230/232/234/236/238	140	173/181/187	-	96
H201F1.17	228/230/232/234/236/238	140	173/181/187	-	96
H201F1.18	228/230/232/234/236/238	140	173/181/187	-	96
H202P1	-	140/147	165/173/177	-	96
H202P2	Not available				

H202F1.1	228/230/232/234/236/238	140/151	173/181/189	-	96
H202F1.2	-	140/147	165/173/177	-	96
H202F1.3	228/230/232/234/236/238	140/151	173/181/189	-	96
H202F1.4	228/230/232/234/236/238	140/151	173/181/189	-	96
H202F1.5	228/230/232/234/236/238	140/151	173/181/189	-	96
H202F1.6	228/230/232/234/236/238	140/151	173/181/189	-	96
H202F1.7	228/230/232/234/236/238	140/151	173/181/189	-	96
H202F1.8	228/230/232/234/236/238	140/151	173/181/189	-	96
H202F1.9	228/230/232/234/236/238	140/151	173/181/189	-	96
H202F1.10	228/230/232/234/236/238	140/151	173/181/189	-	96
H202F1.11	228/230/232/234/236/238	140/151	173/181/189	-	96
H202F1.12	228/230/232/234/236/238	140/151	173/181/189	-	96
J122P1	Not available				
J122P2	Not available				
J122F1.1	-	140/151	165/173/177/181/196/200	211/213	96/100
J122F1.2	217	140/151	165/173/177/181/196/200	211/213	96/100
J122F1.3	217	140/151	165/173/177/181/196/200	211/213	96/100
J124P1	232/234/236	138/140/151/157	165/173/181/185/190/196	211/213	96
J124P2	Not available				
J124F1.1	232/234/236	138/140/151/157	165/173/181/185/190/196	211/213	96
J124F1.2	232/234/236	138/140/151/157	165/173/181/185/190/196	211/213	96
J124F1.3	232/234/236	138/140/151/157	165/173/181/185/190/196	211/213	96
J124F1.4	232/234/236	138/140/151/157	165/173/181/185/190/196	211/213	96
J124F1.5	-	140	173/177/194	211/213	96
J124F1.6	232/234/236	138/140/151/157	165/173/181/185/190/196	211/213	96
J124F1.7	232/234/236	138/140/151/157	165/173/181/185/190/196	211/213	96
J124F1.8	217	140	173/177/194	211/213	96
J124F1.9	232/234/236	138/140/151/157	165/173/181/185/190/196	211/213	96
J130P1	230/232/234/238	140/153	173/177/190/194	211/213	96
J130P2	Not available				
J130F1.1	-	140/153	173/177/190/194	211/213	96
J130F1.2	230/232/234/238	140/153	173/177/190/194	211/213	96
J130F1.3	230/232/234/238	140/153	173/177/190/194	211/213	96
J130F1.4	230/232/234/238	140/153	173/177/190/194	211/213	96
J130F1.5	230/232/234/238	140/153	173/177/190/194	211/213	96
J130F1.6	230/232/234/238	140/153	173/177/190/194	211/213	96
J130F1.7	230/232/234/238	140/153	173/177/190/194	211/213	96
J130F1.8	230/232/234/238	140/153	173/177/190/194	211/213	96
J130F1.9	230/232/234/238	140/153	173/177/190/194	211/213	96
J130F1.10	230/232/234/238	140/153	173/177/190/194	211/213	96
J130F1.11	230/232/234/238	140/153	173/177/190/194	211/213	96
J130F1.12	230/232/234/238	140/153	173/177/190/194	211/213	96
J202P1	Not available				
J202P2	Not available				
J202F1.1	217/227/229/231/235/247	140/151	177/194	-	96
J202F1.2	217/227/229/231/235/247	140/151	177/194	-	96
