# Supplementary material for: Variation in gene expression within clones of the earthworm Dendrobaena octaedra
Source: PLoS One. 2017 Apr 6;12(4):e0174960. doi: 10.1371/journal.pone.0174960 (PMC5383104; doi:10.1371/journal.pone.0174960)
Supplement: S4 Table — Pairwise comparisons for differences in EV were performed using LSD post hoc test. (PDF) [file pone.0174960.s004.pdf]

S4 Table. Differences in the estimate of variation values (EV) between each group in the study design, including the within families group, in the offspring dataset. Pairwise comparisons for differences in EV were performed using LSD post hoc test.

	Within individuals		Within genotypes	
	Mean difference	p value	Mean difference	p value
Within families	0.720	0.016*	0.197	0.237
Within genotypes	0.917	0.014*	-	-
Over all genotypes	1.197	0.020*	0.280	0.157
