# Supplementary material for: Variation in gene expression within clones of the earthworm Dendrobaena octaedra
Source: PLoS One. 2017 Apr 6;12(4):e0174960. doi: 10.1371/journal.pone.0174960 (PMC5383104; doi:10.1371/journal.pone.0174960)
Supplement: S5 Table — Difference in the Estimate of Variation (EV) between different groups (within individuals, within families [in offspring data only], within genotypes and over all genotypes) for each gene separately, and pairwise comparisons between groups: 1. comparison is between within individuals and within families, 2. comparison is between within families and within genotypes for offspring data and within individuals and within genotypes for parent data, and 3. comparison is between within genotypes and over all genotypes. Bartlett test was used for the overall analysis and a F-test was used for pairwise comparisons. (PDF) [file pone.0174960.s005.pdf]

S5 Table. Difference in the estimate of variation (EV) between different groups (within individuals, within families [in offspring data only], within genotypes and over all genotypes) for each gene separately, and pairwise comparisons between groups: 1. comparison is between within individuals and within families, 2. comparison is between within families and within genotypes for offspring data and within individuals and within genotypes for parent data, and 3. comparison is between within genotypes and over all genotypes. Bartlett test was used for the overall analysis and a F-test was used for pairwise comparisons.

		Overall			pairwise comparison 1		pairwise comparison 2		pairwise comparison 3	
		df	χ^2	P	F	P	F	P	F	P
Parents	AkRed	2	66.23	<0.001	-	-	5.48	<.0001	1.43	0.070
	CarRed	2	67.65	<0.001	-	-	1.98	0.008	3.66	<0.001
	ChitDo	2	25.25	<0.001	-	-	2.86	<0.001	1.16	0.272
	ChymInh	2	44.01	<0.001	-	-	2.52	<0.001	1.97	0.002
	Dehyd	2	91.99	<0.001	-	-	12.06	<0.001	1.55	0.056
	Fuco	2	69.84	<0.001	-	-	4.24	<0.001	1.91	0.003
	Leuc	2	48.13	<0.001	-	-	2.07	0.001	2.46	<0.001
	Pyr	2	84.82	<0.001	-	-	1.52	0.061	4.80	<0.001
	Xyl	2	56.86	<0.001	-	-	4.54	.0001	1.82	0.012
	HSP40	2	36.23	<0.001	-	-	3.44	<0.001	1.42	0.083
	HSP70	2	49.97	<0.001	-	-	4.24	<0.001	1.32	0.118
	MT	2	122.00	<0.001	-	-	6.71	<0.001	2.61	<.0001
OffSpring	AkRed	3	10,03	0.007	2.38	0.006	1.02	0.475	1.21	0.261
	ChitDo	3	47.25	<0.001	4.64	<0.001	0.98	0.523	1.05	0.413
	ChymInh	3	114.26	<0.001	3.67	<0.001	1.96	0.001	1.59	0.017
	Dehyd	3	66.76	<0.001	6.62	<0.001	1.26	0.184	1.00	0.495
	MT	3	129.69	<0.001	12.55	<0.001	1.03	0.444	1.34	0.094
