# Supplementary material for: Variation in gene expression within clones of the earthworm Dendrobaena octaedra
Source: PLoS One. 2017 Apr 6;12(4):e0174960. doi: 10.1371/journal.pone.0174960 (PMC5383104; doi:10.1371/journal.pone.0174960)
Supplement: S6 Table — Kruskal Wallis analysis for the parents and Mann-Whitney test for the offspring. (PDF) [file pone.0174960.s006.pdf]

S6 Table. Differences in gene expression of each gene between different genotypes. Kruskal Wallis analysis for the parents and Mann-Whitney test for the offspring.

		df	X^2	p
Parents	AkRed	5	9.93	0.077
	CarRed	5	17.34	0.004
	ChitDo	5	6.25	0.283
	ChymInh	5	8.72	0.121
	Dehyd	5	7.68	0.175
	Fuco	5	12.46	0.029
	Leuc	5	15.15	0.010
	Pyr	5	11.25	0.047
	Xyl	5	12.24	0.032
	HSP40	5	13.16	0.022
	HSP70	5	11.02	0.051
	MT	5	14.63	0.012
Offspring	AkRed	1	23.00	<0.001
	ChitDo	1	59.00	0.026
	ChymInh	1	15.00	<0.001
	Dehyd	1	96.00	0.512
	MT	1	31.00	<0.001
