# Supplementary material for: Variation in gene expression within clones of the earthworm Dendrobaena octaedra
Source: PLoS One. 2017 Apr 6;12(4):e0174960. doi: 10.1371/journal.pone.0174960 (PMC5383104; doi:10.1371/journal.pone.0174960)

S1 Fig. Estimate of variation (EV) in the different groups (within individuals, within families, within genotypes and over all genotypes) in the offspring dataset. Box plots show the median EV for all target genes (n=5) and 25% upper and lower quartiles as well as minimum and maximum.

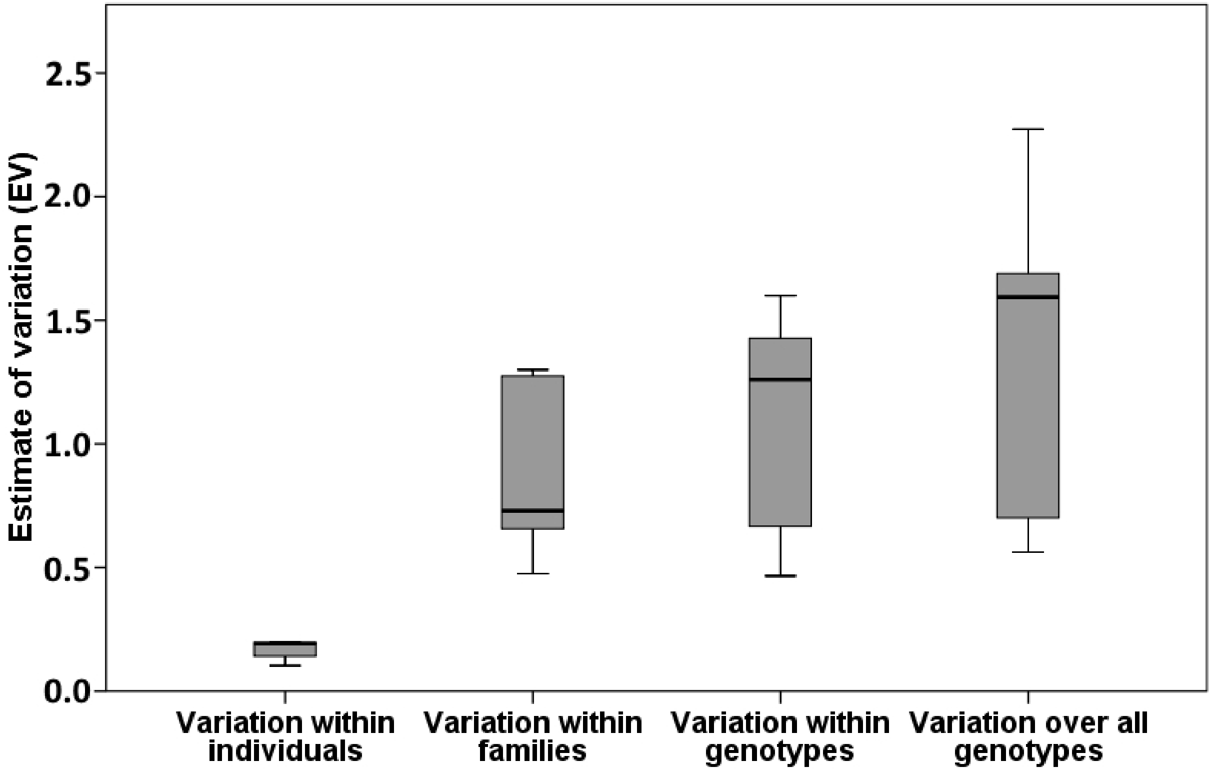

Supplement: S1 Fig — Box plots show the median EV for all target genes (n = 5) and 25% upper and lower quartiles as well as minimum and maximum. (PDF) [file pone.0174960.s007.pdf]
