# Supplementary material for: Variation in gene expression within clones of the earthworm Dendrobaena octaedra
Source: PLoS One. 2017 Apr 6;12(4):e0174960. doi: 10.1371/journal.pone.0174960 (PMC5383104; doi:10.1371/journal.pone.0174960)

S2 Fig. Estimate of variation (EV) in the different groups (within individuals, within genotypes and over all genotypes) for the parent data when using only the subset of five genes (AkRed, ChitDo, ChymInh, Dehyd and MT). Box plot shows all the EV values calculated for each gene separately for each of the groups (median, 25% upper and lower quartile, minimum, maximum, and outliers).

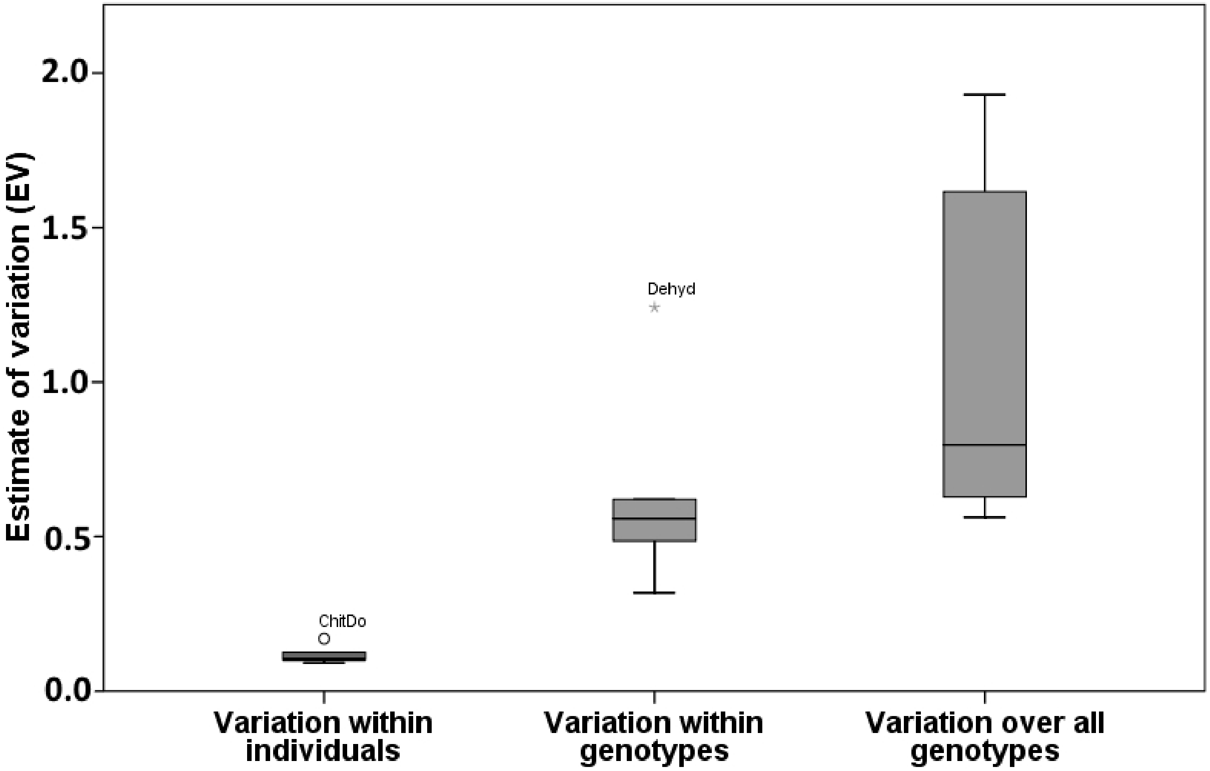

Supplement: S2 Fig — Box plot shows all the EV values calculated for each gene separately for each of the groups (median, 25% upper and lower quartile, minimum, maximum, and outliers). (PDF) [file pone.0174960.s008.pdf]
