# Supplementary material for: Variation in gene expression within clones of the earthworm Dendrobaena octaedra
Source: PLoS One. 2017 Apr 6;12(4):e0174960. doi: 10.1371/journal.pone.0174960 (PMC5383104; doi:10.1371/journal.pone.0174960)

S3 Fig. Estimate of variation in all genes (AkRed, CarRed, ChitDo, ChymInh, DeHyd, Fuco, Leuc, Pyr, Xyl, HSP40, HSP70 and MT) within parent genotypes (H1, H2, H3, J1, J2 and J3). Box plot shows all the EV values calculated for each gene separately for each of the genotypes (median, 25% upper and lower quartile, minimum, maximum, and outliers).

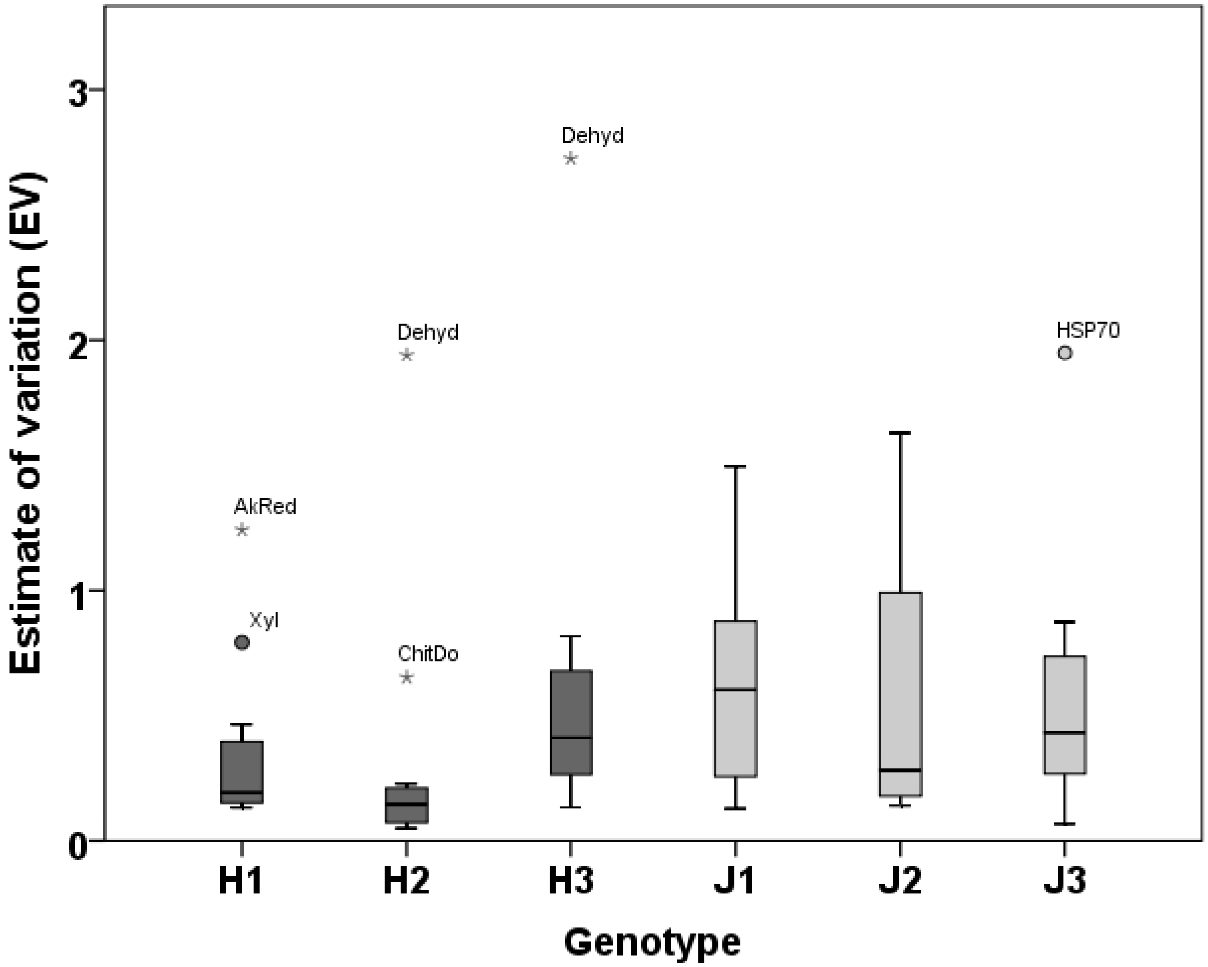

Supplement: S3 Fig — Box plot shows all the EV values calculated for each gene separately for each of the genotypes (median, 25% upper and lower quartile, minimum, maximum, and outliers). (PDF) [file pone.0174960.s009.pdf]
