# Supplementary material for: Variation in gene expression within clones of the earthworm Dendrobaena octaedra
Source: PLoS One. 2017 Apr 6;12(4):e0174960. doi: 10.1371/journal.pone.0174960 (PMC5383104; doi:10.1371/journal.pone.0174960)

S4 Fig. Estimate of variation (EV) for two genotypes, H1 and J1, in the offspring dataset. Box plot shows all the EV values calculated for each gene separately for each genotype (median, 25% upper and lower quartile, minimum, and maximum).

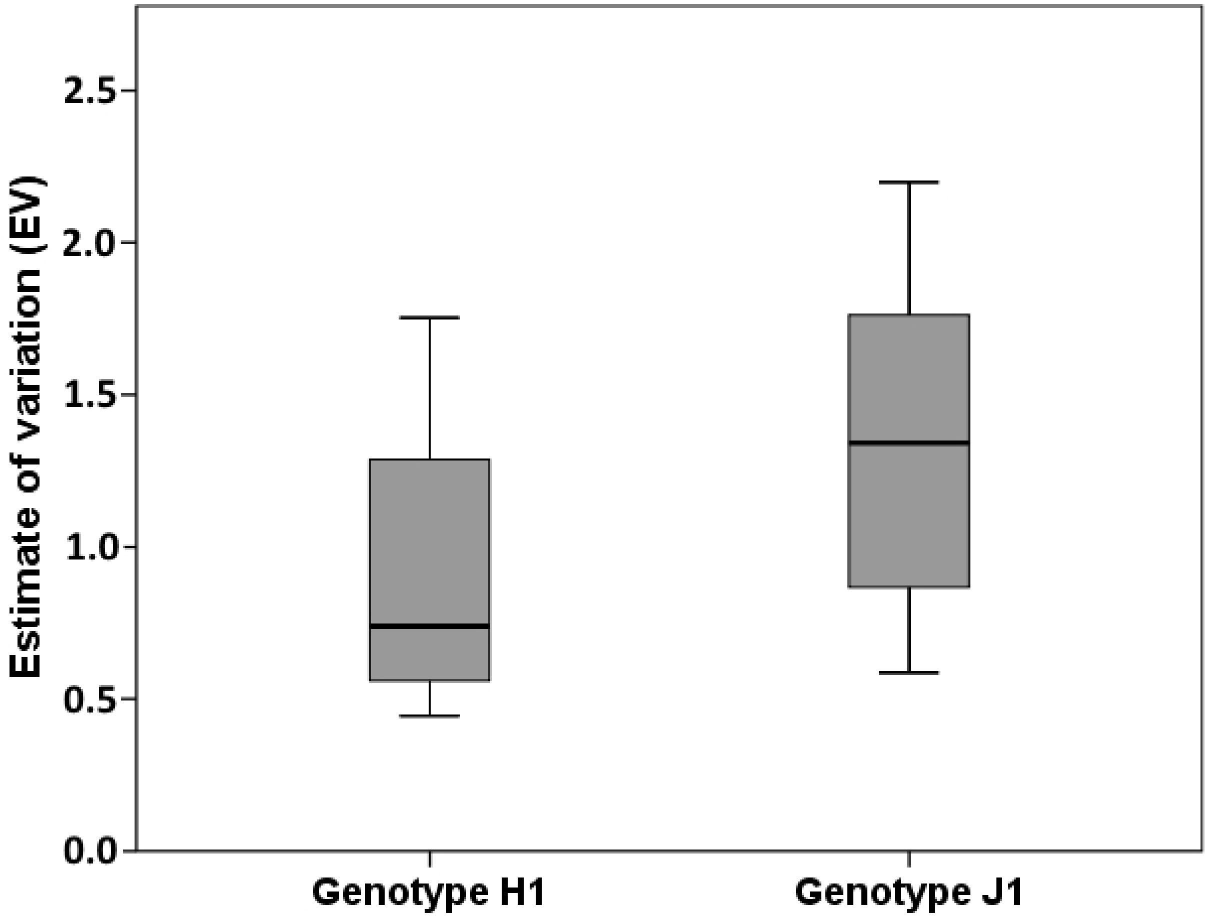

Supplement: S4 Fig — Box plot shows all the EV values calculated for each gene separately for each genotype (median, 25% upper and lower quartile, minimum, and maximum). (PDF) [file pone.0174960.s010.pdf]
