# Supplementary material for: Variation in gene expression within clones of the earthworm Dendrobaena octaedra
Source: PLoS One. 2017 Apr 6;12(4):e0174960. doi: 10.1371/journal.pone.0174960 (PMC5383104; doi:10.1371/journal.pone.0174960)

S5 Fig. Gene expression (RQ) of each gene in each parent genotype. Box plot shows all the normalized relative quantity (RQ) values for each genotype (median, 25% upper and lower quartile, minimum, maximum, and outliers).

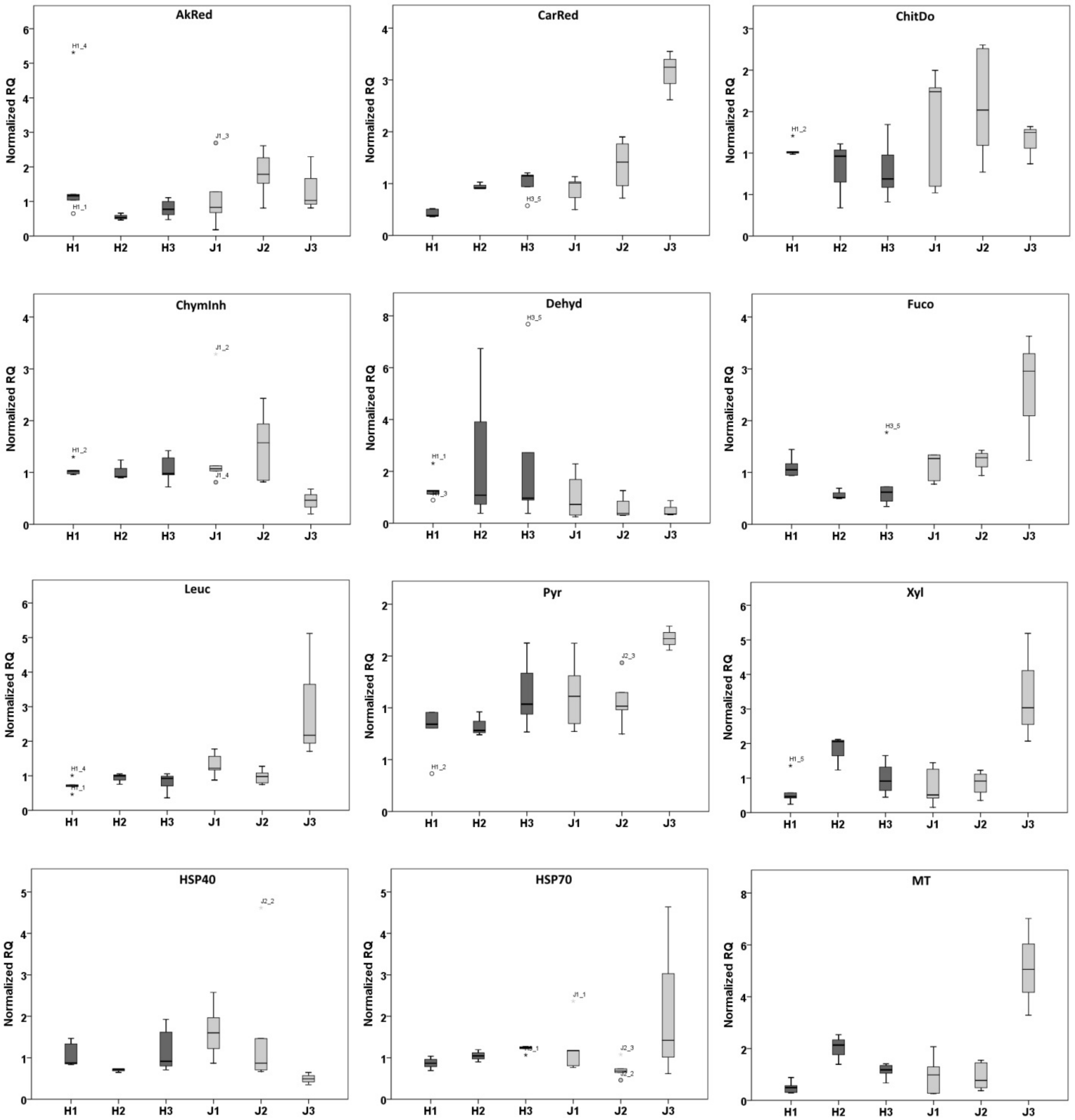

Supplement: S5 Fig — Box plot shows all the normalized relative quantity (RQ) values for each genotype (median, 25% upper and lower quartile, minimum, maximum, and outliers). (PDF) [file pone.0174960.s011.pdf]
